# Impact of qualitative, semi-quantitative, and quantitative analyses of dynamic contrast-enhanced magnet resonance imaging on prostate cancer detection

**DOI:** 10.1371/journal.pone.0249532

**Published:** 2021-04-05

**Authors:** Farid Ziayee, Tim Ullrich, Dirk Blondin, Hannes Irmer, Christian Arsov, Gerald Antoch, Michael Quentin, Lars Schimmöller

**Affiliations:** 1 Medical Faculty, Department of Diagnostic and Interventional Radiology, Univ Dusseldorf, Dusseldorf, Germany; 2 Medical Faculty, Department of Urology, Univ Dusseldorf, Dusseldorf, Germany; Medical University of Vienna, AUSTRIA

## Abstract

Dynamic contrast enhanced imaging (DCE) as an integral part of multiparametric prostate magnet resonance imaging (mpMRI) can be evaluated using qualitative, semi-quantitative, or quantitative assessment methods. Aim of this study is to analyze the clinical benefits of these evaluations of DCE regarding clinically significant prostate cancer (csPCa) detection and grading. 209 DCE data sets of 103 consecutive patients with mpMRI (T2, DWI, and DCE) and subsequent MRI-(in-bore)-biopsy were retrospectively analyzed. Qualitative DCE evaluation according to PI-RADS v2.1, semi-quantitative (curve type; DCE score according to PI-RADS v1), and quantitative Tofts analyses (K^trans^, k_ep,_ and v_e_) as well as PI-RADS v1 and v2.1 overall classification of 209 lesions (92 PCa, 117 benign lesions) were performed. Of each DCE assessment method, cancer detection, discrimination of csPCa, and localization were assessed and compared to histopathology findings. All DCE analyses (p<0.01–0.05), except v_e_ (p = 0.02), showed significantly different results for PCa and benign lesions in the peripheral zone (PZ) with area under the curve (AUC) values of up to 0.92 for PI-RADS v2.1 overall classification. In the transition zone (TZ) only the qualitative DCE evalulation within PI-RADS (v1 and v2.1) could distinguish between PCa and benign lesions (p<0.01; AUC = 0.95). None of the DCE parameters could differentiate csPCa from non-significant (ns) PCa (p ≥ 0.1). Qualitative analysis of DCE within mpMRI according to PI-RADS version 2.1 showed excellent results regarding (cs)PCa detection. Semi-quantitative and quantitative parameters provided no additional improvements. DCE alone wasn’t able to discriminate csPCa from nsPCa.

## Introduction

Prostate cancer (PCa) shows increased perfusion compared to normal tissue due to higher micro-vessel density, arteriovenous shunts, and higher vascular permeability. Therefore, dynamic contrast enhanced MRI (DCE) represents an additional tool for the detection of PCa, especially in the peripheral zone (PZ) [1]. Since the 2015 updated Prostate Imaging Reporting and Data System (PI-RADS) version (version 2), focal early or contemporaneous enhancement in DCE of a suspicious lesion (in T2/DWI) can upgrade lesions from PI-RADS overall assessment category 3 to 4 unless it demonstrates features of benign prostatic hyperplasia (BPH) on T2-weighted images [2]. The benefit of a qualitative DCE analysis is its relatively simple and timesaving usage. However, this approach represents a subjective judgment of the reader and the gathered information of the exact perfusion is limited [3,4].

A semi-quantitative approach as implemented in the first version of PI-RADS requires analysis of the concentration time-curve assigned to three different types with distinct likelihoods of the presence of PCa [5]. The curve type provides information about the in- and outflow of contrast media (CM) into the tissue over time.

Based on pharmacokinetic models, such as the extended two-compartment model by Tofts et al., analysis of perfusion parameters (K^trans^, k_ep_, and v_e_) allows the exact quantification of the CM flowrate into the tissue and back to the intravascular compartment after assessment of the arterial input function. The transfer constants K^trans^ and k_ep_ characterize the transfer rate of CM between the intravascular and the extravascular extracellular space whereas v_e_ represent the fractional extravascular extracellular space volume. As a quantitative method with absolute values, this approach permits a direct comparison of perfusion parameters (K^trans^, k_ep,_ and v_e_) in tumor, physiological peripheral (PZ) or transition zone (TZ) tissue [6]. This method requires specific software assistance, additional time, and expertise. Although the quantitative and semi-quantitative analyses are of great interest in scientific investigation, the additional benefit in clinical routine is still controversial. As far as we know, there was no intra-individual comparison of all three DCE assessment methods available in the literature at the time of this study.

To avoid overdiagnosis and overtreatment, there is a need to identify clinically significant (cs) PCa, mostly defined as International Society of Urological Pathology (ISUP) Grade Group ≥ 2 (Gleason score of ≥3+4 = 7), since significant tumors require timely treatment while low-grade tumors can be monitored within active surveillance [7,8]. Post therapeutic complications and complaints after treatment of insignificant disease represent a major problem [9]. Therefore, it is not only of scientific but also of clinical interest to investigate if DCE is able to distinguish between csPCa and non-significant (ns) PCa.

The primary objective of this study was to compare qualitative, semi-quantitative, and quantitative DCE assessment methods in order to evaluate the significance for detection of csPCa.

## Materials and methods

### Study design

This retrospective cohort study was conducted in the Department of Diagnostic and Interventional Radiology and the Department of Urology at the University Hospital Düsseldorf (Medical Faculty, Heinrich-Heine-University, Düsseldorf, Germany) from August 2015 to May 2020. All data were fully anonymized before been accessed. The study has been approved by the local institutional review board (Ethical Review Committee, Medical Faculty, Heinrich-Heine-University Düsseldorf; study number 3612). All patients signed a written informed consent for their medical records/images to be used in research. Refusal to take part in the study did not influence patients care.

Consecutive patients with standard-of-care mpMRI prostate examinations with suspected PCa and subsequent MR-(in-bore)-biopsy plus systematic 12-core TRUS-guided biopsy between January and December 2012 were retrospectively included. All patients had elevated PSA-levels >4 ng/ml, no known prostate tumor, and no contraindications to MRI or prostate biopsy. All patients with a PI-RADS v1 overall classification of 4 or 5 had received a targeted MR-(in-bore)-biopsy of every discribed lesion in the MRI report (independent of the PI-RADS v1 lesion score). The majority of patients were previously enrolled in prospective randomized trials comparing MR-(in-bore)-biopsy to MRI-ultrasound fusion or systematic transrectal ultrasound guided prostate biopsy either in patients after initial biopsy or in biopsy biopsy-naïve men (ClinicalTrials.gov identifier: NCT02220517, NCT01553838). Diagnostic performance of mpMRI and MRI-guided biopsy has been reported earlier [10,11] and is not part of this study.

Different DCE methods of all histologically confirmed lesions were retrospectively analyzed in consensus by two experienced uroradiologists with more than 10 years of experience in reading prostate MRI (LS and MQ).

The distinct DCE methods were compared in matter of their differentiation between PCa and benign lesions and between csPCa with an ISUP Grade Group ≥ 2 (Gleason score ≥3+4 = 7) and nsPCa with an ISUP Grade Group 1. Patient’s age, PSA values, biopsy history, PI-RADS scoring, ADC values, biopsy results including localization, quantity of lesions, and Gleason scoring were assessed. Lesion size was measured in three diameters in the Picture Achieving and Communicating System (PACS; Sectra Imtec AB, IDS7, Sweden). Measurements were performed on the sequence that shows the lesion best, usually T2-weighted images or ADC (especially for peripheral lesions).

### Image acquisition

Images were acquired with a 3 Tesla (T) MRI scanner (Magnetom TIM Trio Systems, Siemens Healthcare GmbH, Germany) using a six-channel phased-array body combined with 32-channel spine coil. The mp-MRI protocol included T2 (axial, sagittal, and coronal), T1, DWI, and DCE (S1 Table): axial T2-weighted turbo spin-echo sequences (TR 10,630 ms, TE 117 ms, field of view (FOV) 12.8 cm, slice thickness 3.0 mm), axial T1-weighted turbo spin-echo images (TR 650 ms, TE 13 ms, FOV 30 cm, slice thickness 5.0 mm), and axial diffusion weighted imaging with a singleshot spin-echo echo-planar sequence (TR 4,600 ms, TE 90 ms, FOV 20.4 cm, slice thickness 3.0 mm) using five b-values (0, 250, 500, 750, 1000 s/mm^2^). The T1 volumetric interpolated breath-hold examination (VIBE) DCE sequence (TR 5.26 ms, TE 1.76 ms, flip angle 12°) was conducted with a field of view (FoV) of 192x192 mm, a resolution of 128x128 pixels, and a temporal resolution of 9.8s. Voxel size was 3 mm with no gap. In total 31 dynamic scans were acquired. Acquisition time was 5:05 minutes. Gadolinium based contrast agent Gd-DOTA (Dotarem R, Guerbet, France) was injected after the initial native measurements with a flow of 3 ml/s and a dosage of 0.1 mmol/Kg body weight followed by a 50 ml flush of NaCl. Patients received butylscopolamine (20 mg Buscopan^®^, Boehringer Ingelheim Pharma, Ingelheim, Germany) to suppress bowel peristalsis. No additional rectal preparation was performed.

### MR-guided biopsy

MRI-(in-bore)-biopsy was performed transrectally with the same MRI system in prone position by experienced uroradiologists (LS and MQ) [12]. Images for biopsy planning were obtained using sagittal and transverse T2-haste sequences (TR 2000 ms; TE 76 ms; FOV 28 cm; slice thickness 3.0 mm). Image data was transferred to a workstation (DynaCAD, Invivo, Philips Healthcare, USA) for biopsy planning. A needle-in control scan was performed in two different planes to ensure correct needle placement. At least two samples per lesion were gathered with an MR compatible 18-gauge biopsy-gun (Invivo). Afterwards, an additional systematic 12-core transrectal ultrasound-guided biopsy was performed by an experienced urologist blinded to the MRI report with an 18-gauge biopsy gun (Bard Medical, Karlsruhe, Germany).

### Qualitative DCE analysis

Qualitative analysis was conducted retrospectively according to the PI-RADS v2.1 criteria [3]. The focal early or contemporaneous enhancement of a suspicious lesion in T2W and/or DWI was considered as positive DCE PI-RADS v2.1 single score. Additionally, a PI-RADS v2.1 overall lesion classification using all mpMRI information with respect to the dominant sequence correlated to the lesion localization (peripheral or transition zone) was determined retrospectively.

### Semiquantitative DCE analysis

Mean signal intensities (SI) of all pixels within a region of interest (ROI) were integrated in a SI-curve. The curve was converted into a CM-time-curve (CTC) using DynaCAD^®^. The CTC was afterwards assigned to one of three different curve types (progressive curve type, plateau curve type, and wash-in/wash-out curve type) as implemented in PI-RADS v1. In addition to the curve-typing, DCE PI-RADS v1 single score and PI-RADS v1 overall lesion classification were also determined [5].

### Quantitative DCE analysis

Perfusion maps for K^trans^, k_ep_, and v_e_ were generated by the commercially available software DynaCAD^®^ using the customized DCE sequence parameters (magnet strength, flip angle, repetition time, number of phases, CM arrival delay, CM dose, injection duration, enhancement threshold, and fit coefficient). DynaCAD^®^ uses a population-based arterial input function (AIF) [13]. Quantitative perfusion parameters K^trans^ and *k*_ep_ for all lesions were determined in the corresponding perfusion map by ROI based measurements (in min^-1^). *ve* was calculated as quotient of K^trans^ and *k*_ep_.

### Histopathology

All biopsy samples were evaluated by an experienced pathologist following the recommendations of the International Society of Urological Pathology (ISUP) [14]. For this study an ISUP Grade Group ≥ 2 (Gleason score ≥3+4 = 7) was considered as csPCa. Reference standard for pathology was the targeted MR-(in-bore)-biopsy of each lesion.

### Statistics

Statistical analysis was performed with SPSS 21 (IBM, Armonk, New York, USA). Results are presented with mean value and standard deviation (SD) or median and interquartile range (IQR). Mann-Whitney-U-test was employed for unpaired samples. A two-tail p-value ≤0.05 was considered significant. Receiver operating characteristic (ROC) analysis was conducted. AUC values were classified excellent (0.90–1), good (0.80–0.90), fair (0.70–0.80), poor (0.60–0.70), and/or fail (0.50–0.60).

## Results

### Study population

One hundred three patients were included in this study. PCa was verified by biopsy in 92 lesions of 53 patients. ISUP grades were as folows: ISUP 1: 18 lesions of 8 patients, ISUP 2: 54 lesions of 30 patients, ISUP 3: 13 lesions of 11 patients, ISUP 4: 3 lesions of 2 patients, and ISUP 5: 4 lesions of 2 patients. 119 lesions were located in TZ, 75 in PZ, 15 in the AFS/CZ (anterior fibromuscular struma or central zone). 117 lesions and 50 patients showed negative biopsy results. Age, PSA-values, prostate volume, and PSA-density with corresponding p-values are shown in Table 1.

**Table 1:** Baseline characteristics.

	all	PCa	no PCa	p-value
**Patients**	103	53	50	-
**MRI lesions**	209	92	117	-
**Age** (mean ± SD)	67 ± 7.5	68 ± 7.7	66 ± 7.2	0.1
**PSA** (median—IQR)	7.7 (5.8–10)	8.1 (6–11)	7.1 (5.6–10)	0.3
**PV** (median—IQR)	47 (36–71)	45 (33–62)	58 (40–84)	**0.01**
**PSAD** (median—IQR)	0.15 (0.11–0.22)	0.17 (0.12–0.32)	0.13 (0.10–0.18)	**<0.01**

PV = Prostate volume; PSAD = PSA density; IQR = interquartile range; SD = standard deviation; p-values ≤0.05 are considered to be significant and are given in bold.

### Qualitative, semi-quantitative, and quantitative DCE analysis

#### All lesions

PI-RADS single score for DCE (v1 and v2.1) and PI-RADS overall classification (v1 and v2.1) per lesion were able to differentiate PCa (n = 92) from benign (n = 117) lesions with p<0.01, whereas curve type and quantitative DCE analysis did not provide statistically significant results. In ROC analysis PI-RADS v2.1 overall classification demonstrated the highest area under the curve (AUC = 0.94), whereas among the other DCE analysis AUC of DCE v2.1 was highest (AUC = 0.91).

#### Peripheral zone (PZ) lesions

**Fig 1:**
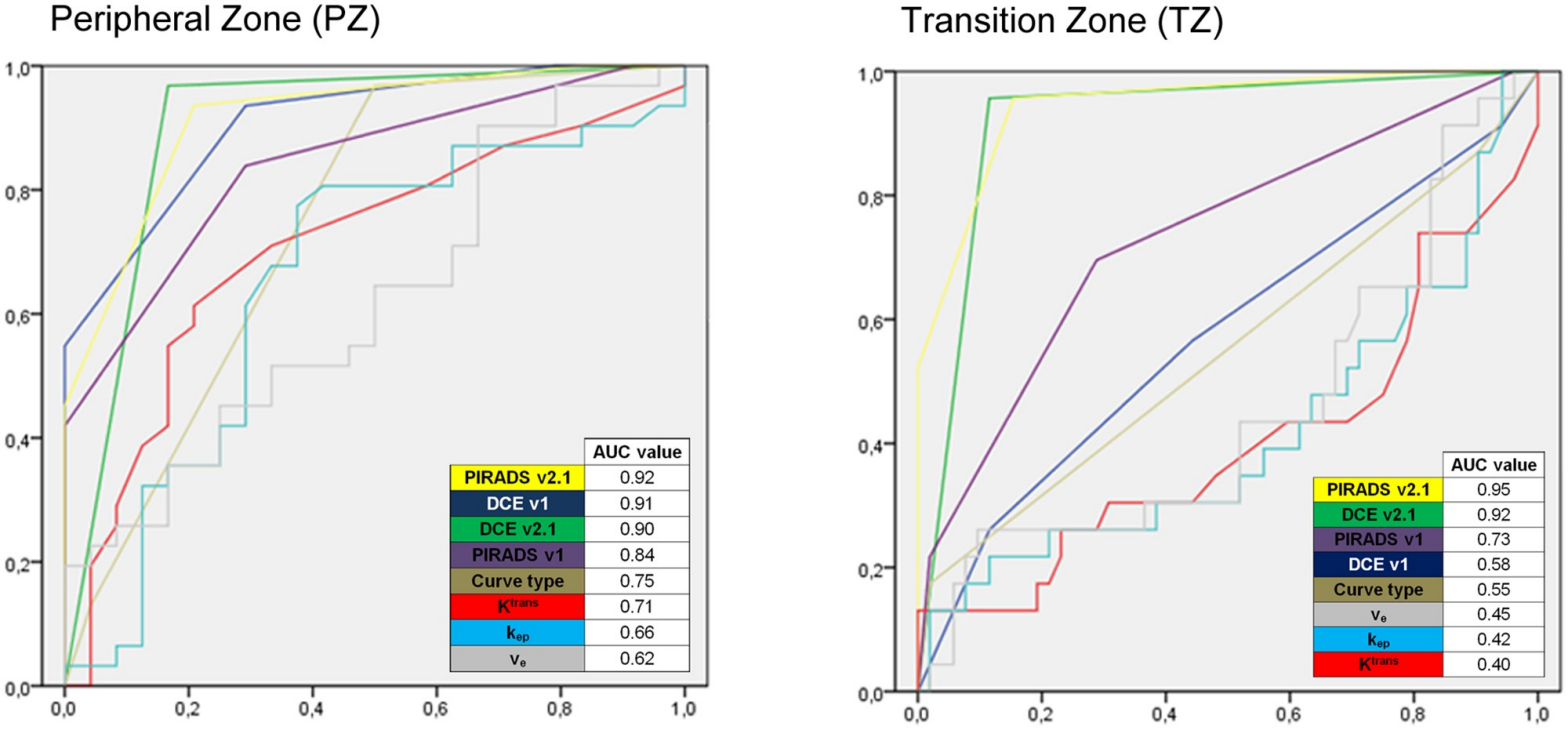
PI-RADS v2.1 shows excellent performance in assessing DCE. ROC-analysis of qualitative (DCE v2.1), semiquantative (DCE v1 and curve type), and quantitative (K^trans^, k_ep_, v_e_) DCE parameters and multiparametric PI-RADS v1 and PI-RADS v2.1 for differentiation of prostate cancer and benign lesions in the peripheral zone (PZ, right) and transition zone (TZ, left). Areas under the curve (AUC) values are given in the bottom of each plot.

**Fig 2:**
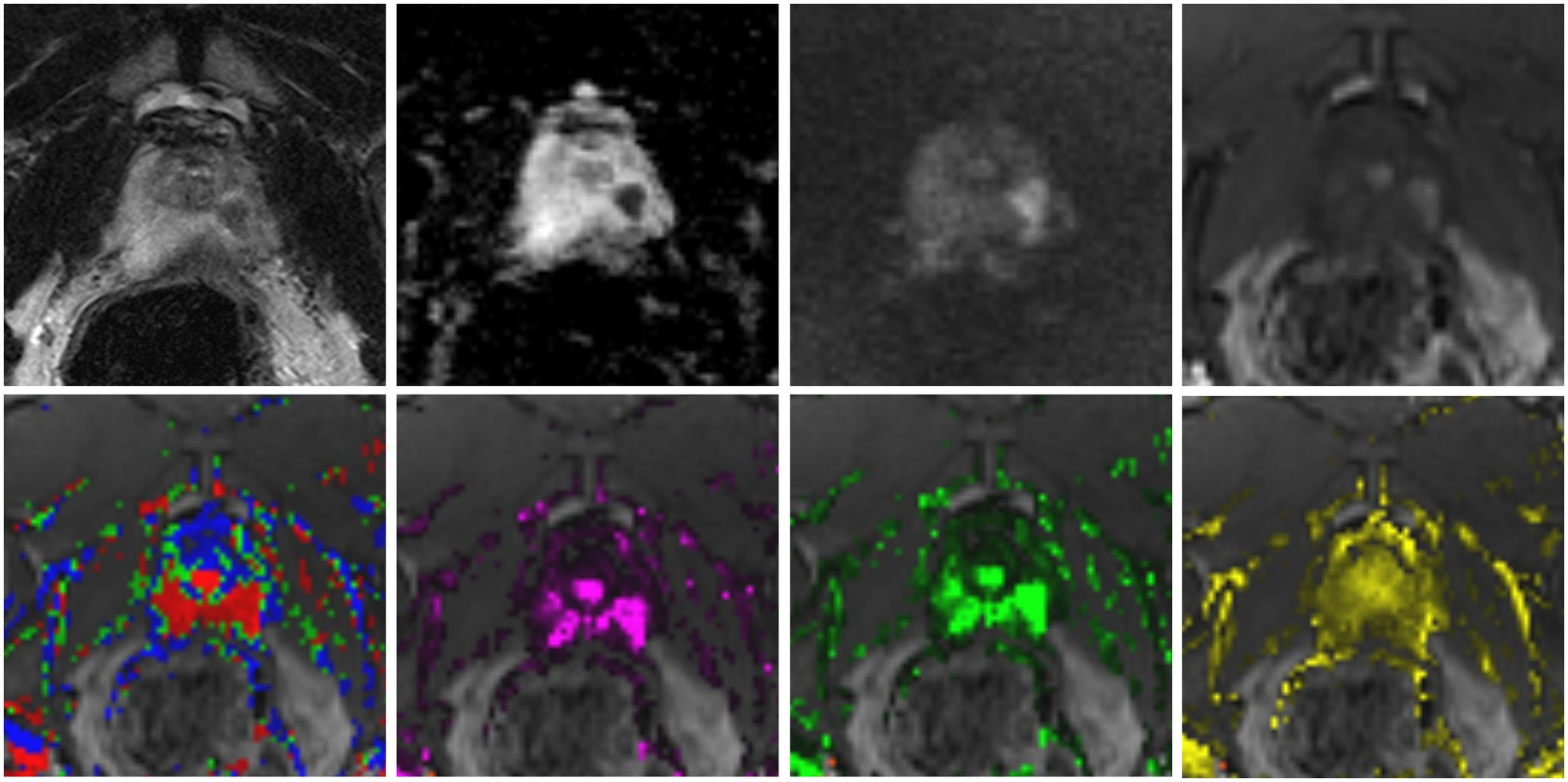
Peripheral PCa lesion is declinable in most of the perfusion maps. Example of a peripheral PCa lesion. *Upper row*: multiparametric MRI (from left to right with T2, DWI, ADC, and DCE showing a left peripheral carcinoma with gleason score 4+3 = 7 in a 48 year old patient. *Lower row*: corresponding perfusion maps (from left to right) colour-coded, K^trans^, k_ep_, and v_e_.

All parameters (qualitative, semiquantitative, and quantitative DCE analysis including multiparametric analysis with PIRADS overall classification v1 and v2.1), except v_e_ were statistically different between PCa and benign lesions in PZ with highest AUC in ROC for PI-RADS v2.1 (AUC = 0.92), followed by DCE v1 (AUC = 0.91) and DCE v2.1 (AUC = 0.90) (Table 2, Figs 1 and 2).

**Table 2:** Peripheral zone (PZ): Comparative analyses of qualitative (DCE v2.1), semiquantative (DCE v1 and curve type), and quantitative (K^trans^, k_ep_, v_e_) DCE parameters and multiparametric PI-RADS v2.1 and PI-RADS v1 in PZ lesions with and without prostate carcinoma (PCa).

PZ	PCa (n = 48)	benign (n = 27)	p-value
**PI-RADS**_**v2.1**_ (median—IQR)	4 (4–5)	3 (3–3)	**<0.01**
**DCE**_**v2.1**_ (median—IQR)	1 (1–1)	0 (0–0)	**<0.01**
**PI-RADS**_**v1**_ (median—IQR)	5 (4–5)	3 (3–4)	**<0.01**
**DCE**_**v1**_ (median—IQR)	4 (3–4)	2 (2–3)	**<0.01**
**Curve type** (median—IQR)	2 (2–2)	2 (1–2)	**<0.01**
**K**^**trans**^ (min^-1^) (mean ± SD)	0.15 ± 0.10	0.9 ± 0.08	**0.001**
**k**_**ep**_ (min^-1^) (mean ± SD)	2.5 ± 1.5	2.0 ± 1.7	**0.05**
**v**_**e**_ (mean ± SD)	0.06 ± 0.02	0.05 ± 0.02	0.2

IQR = interquartile range; SD = standard deviation; p-values ≤0.05 are considered to be significant and are given in bold.

#### Transition zone (TZ) lesions

When comparing PCa and benign lesions in TZ, only values for PI-RADS overall classification (v1 and v2.1) and DCE score (v2.1) were statistically significant with highest AUC in ROC for PI-RADS v2.1 (AUC = 0.95), followed by DCE v2.1 (0.92) and PI-RADS v1 (AUC = 0.73). None of the other DCE methods demonstrated significant results (Table 3, Fig 1).

**Table 3:** Transition zone (TZ): Comparative analyses of qualitative (DCE v2.1), semiquantative (DCE v1 and curve type), and quantitative (K^trans^, k_ep_, v_e_) DCE parameters and multiparametric PI-RADS v2.1 and PI-RADS v1 in TZ lesions with and without prostate carcinoma (PCa).

TZ	PCa (n = 33)	benign (n = 86)	p-value
**PI-RADS**_**v2.1**_ (median—IQR)	5 (4–5)	3 (3–3)	**<0.01**
**DCE**_**v2.1**_ (median—IQR)	1 (1–1)	0 (0–0)	**<0.01**
**PI-RADS**_**v1**_ (median—IQR)	4 (3–5)	3 (3–4)	**<0.01**
**DCE**_**v1**_ (median—IQR)	3 (2–4)	3 (2–3)	0.8
**Curve type** (median—IQR)	2 (2–3)	2 (2–2)	0.3
**K**^**trans**^ (min^-1^) (mean ± SD)	0.13 ± 0.22	0.19 ± 0.12	0.4
**k**_**ep**_ (min^-1^) (mean ± SD)	2.0 ± 2.1	3.5 ± 3.8	0.3
**v**_**e**_ (mean ± SD)	0.05 ± 0.03	0.06 ± 0.03	0.6

IQR = interquartile range; SD = standard deviation; p-values ≤0.05 are considered to be significant and are given in bold.

#### PCa grading

**Fig 3:**
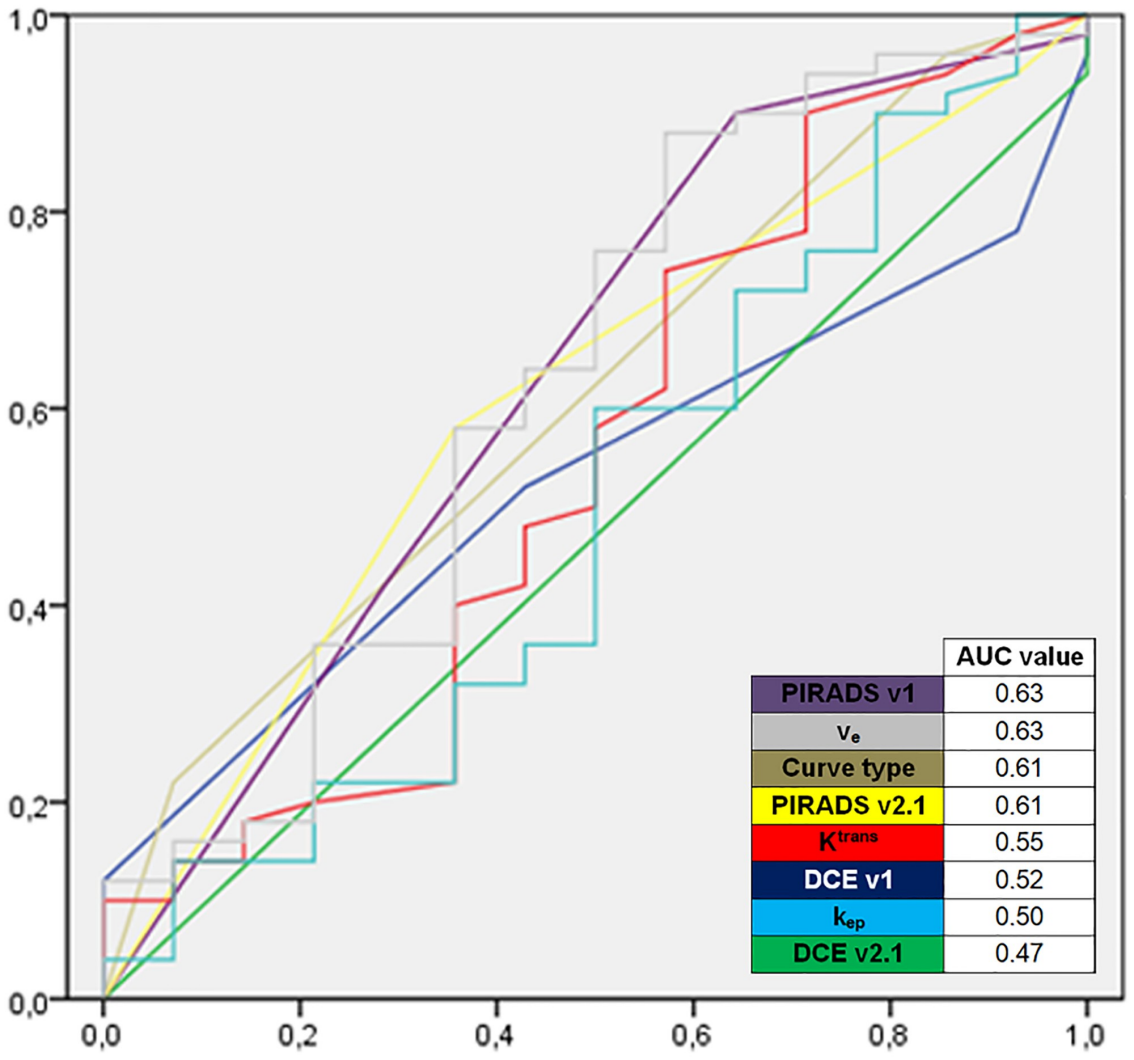
None of the other DCE methods can differentiate low grade tumor from clinicaly significant tumor alone. ROC-analysis of qualitative (DCE v2.1), semiquantative (DCE v1 and curve type), and quantitative (K^trans^, k_ep_, v_e_) DCE parameters and multiparametric PI-RADS v1 and PI-RADS v2.1 for differentiation of clinically significant carcinoma with Gleason score ≥7 vs. insignificant carcinoma with Gleason score <7 Areas under the curve (AUC) are given in the bottom right.

None of the applied methods was able to distinguish csPCa (ISUP Grade Group ≥ 2) from nsPCa (ISUP Grade Group 1) (Table 4). ROC analysis (csPCa vs. nsPCa) showed poor AUC values (highest AUC for PI-RADS (v1) with 0.63) (Fig 3).

**Table 4:** Comparative analyses of qualitative (DCE v2.1), semiquantative (DCE v1 and curve type), and quantitative (K^trans^, k_ep_, v_e_) DCE parameters and multiparametric PI-RADS v2.1 and PI-RADS v1 in all lesions with prostate carcinoma (PCa) vs. benign (left) and all lesions with clinically significant carcinoma (csPCa) with Gleason Score ≥ 7 (ISUP Grade Group ≥ 2) vs. non-significant carcinoma (nsPCa) with Gleason Score 6 (ISUP Grade Group 1) (right).

	csPCa (n = 72)	nsPCa (n = 20)	p-value
**PI-RADS**_**v2.1**_ (median—IQR)	5 (4–5)	4 (4–5)	0.1
**DCE**_**v2.1**_ (median—IQR)	1 (1–1)	1 (1–1)	0.9
**PI-RADS**_**v1**_ (median—IQR)	4 (3–5)	4 (4–5)	0.2
**DCE**_**v1**_ (median—IQR)	4 (3–4)	3 (3–4)	0.8
**Curve type** (median—IQR)	2 (2–2)	2 (2–2)	0.5
**K**^**trans**^ **(min**^**-1**^**)** (mean ± SD)	0.19 ± 0.18	0.14 ± 0.1	0.4
**k**_**ep**_ **(min**^**-1**^**)** (mean ± SD)	2.9 ± 2.1	2.9 ± 2.2	0.9
**v**_**e**_ (mean ± SD)	0.06 ± 0.02	0.05 ± 0.02	0.2

IQR = interquartile range; SD = standard deviation; p-values ≤0.05 are considered to be significant and are given in bold.

## Discussion

Multiparametric MRI analysis according to PI-RADS v2.1 including qualitative DCE evaluation offered excellent diagnostic accuracy for PCa detection. Quantitative and semi-quantitative perfusion parameters were inferior compared to the PI-RADS. No analyzed DCE assessment method was able to significantly differentiate tumor-aggressiveness.

Tan et al. demonstrated that qualitative and semi-quantitative analyses of DCE perform similarly in PCa detection [15]. Chesnais et al. tested eight different semi-quantitative and quantitative DCE parameters for PCa detection in TZ. None of the parameters seemed to demonstrate additional information [16]. Hansford et al. assigned a poor performance of curve type for the differentiation of PCa and benign lesions in PZ [17]. In our study, semi-quantitative perfusion parameters did not show significant differences between PCa and benign lesions in TZ whereas in PZ results were statistically significant, but with lower, fair AUC values in ROC analysis in comparison to qualitative DCE analyses. The fact that both, Type-2 and -3 curves go along with a fast “wash-in” corresponding to a visible early enhancement as implemented in DCE evaluation by PIRADS v2.1 may explain the results. Moreover, semi-quantitative analysis is independent from T2- and DWI whereas the evaluation of DCE in PI-RADS v2.1 requires a suspicious finding in one of the other sequences. Thus, it already includes mpMRI information which represents a major advantage and presumably improves PCa detection rate. This may explain the higher, excellent AUC in ROC analysis for the PI-RADS DCE score. Depending on the micro-vessel density and the leakiness of vessels in the examined tumors all three curve types may occur in PCa (although with different probability) reducing specificity of this method.

In quantitative DCE analysis, none of the three examined parameters demonstrated statistically significant differences between PCa and benign lesions in the TZ. In contrary, K^trans^, and k_ep_ could significantly differentiate PCa in PZ. However, AUC values were only fair. Sanz-Requena et al. [18] and Ocak et al. [19] have also demonstrated the ability of K^trans^ and k_ep_ for the detection of PCa in PZ, while v_e_ failed. Mehrabian et al. confirmed that K^trans^ is appropriate to separate PCa in PZ [20]. Bonekamp et al. could show that DCE ameliorates sensitivity and specificity of cancer detection in PZ, but not in TZ [21]. The quoted literature is consistent with our data. While the additional effort of quantitative DCE analysis in comparison to qualitative evaluation cannot be justified by a better performance in PCa detection objective, absolute values may facilitate comparability and reproducibility of DCE results between facilities and may represent an attractive tool e.g. for therapy monitoring. In contrast, qualitative analyses depend on the reader’s subjective impression and image interpretation.

PI-RADS v2.1 assessment demonstrated excellent results to identify PCa independently of the lesions’ localization. Even though the diagnostic accuracy of prostate mpMRI for PCa detection has been validated in the literature [22,23], more and more authors abandon standard employment of DCE and emphasize a biparametric approach [24]. Although ROC analysis demonstrated a high AUC for the DCE PI-RADS v2.1 single score, the dichotomous nature of this parameter restricts its diagnostic value and the parameter is influenced by multiparametric information.

None of the DCE assessment methods alone was able to distinguish non-significant PCa (ISUP Grade group 1) from clinically significant PCa (ISUP Grade Group ≥ 2). However, mpMRI, as a combination of anatomic and functional imaging, is able to identify csPCa [7]. How far DCE information can contribute to the tumor grading and identifying ISUP Grade Group ≥ 2 cancers would be clinically relevant. Sun et al. found evidence that mpMRI in combination with texture analysis is able to stratify tumor aggressiveness in PCa [25]. Mirak et al. identified quantitative and semi-quantiative DCE parameters as possible contributors to assess tumor aggressiveness [26]. More aggressive PCa may be accompanied by greater probability of tumor-necrosis which may hinder DCE performance and explain our results since necrotic tissue has a low vascularization which reduces SI on DCE imaging for the assessed lesion.

This study has some limitations. Frist, DCE analyses were performed retrospectively only on lesions histologically confirmed using MRI-(in-bore)-biopsy. Therefore, sensitivity, specificity of the DCE, and the question of undetected PCa could not be assessed. Since the study analyzes 92 lesions, including benign and cancer lesions, selection bias is limited. Second, systematic biopsy results were not investigated; therefore the value in non MRI-detected lesions remains unclear. However, only precisely histologically confirmed lesions allowed us to test various DCE metrics on a lesion base analysis. Third, quantitative perfusion parameters were assessed by the DynaCAD software, thus the measured values depend on the software algorithm and the applied AIF. Moreover, there are numerous other semi-quantitative parameters that have not been evaluated in this study. It is possible that a different AIF method or other semi-quantitative parameters would have provided slightly different results. Finally, we did not assess the added value of the different DCE analyses to PI-RADS and special scenarios (e.g. PI-RADS 3).

In conclusion, quantitative and semi-quantitative perfusion analyses do not seem to offer a benefit for PCa detection compared to qualitative analysis. Since mpMRI with qualitative DCE analysis has provided excellent results in PZ and in TZ our data confirm current usage of PIRADS v2.1 standard for PCa detection. Although none of the presented methods could predict tumor aggressiveness, further scientific efforts should be attempted to minimize overdiagnosis of low-grade tumors and associated complications.

## Supporting information

S1 Table MRI-Sequence parameters. (XLS)

## References

[1] Rosenkrantz AB, Sabach A, Babb JS, Matza BW, Taneja SS, Deng FM. Prostate cancer: comparison of dynamic contrast-enhanced MRI techniques for localization of peripheral zone tumor. AJR Am J Roentgenol. 2013;201(3):W471–W478. 10.2214/AJR.12.9737 23971479

[2] Turkbey B, Rosenkrantz AB, Haider MA, Padhani AR, Villeirs G, Macura KJ, et al. Prostate Imaging Reporting and Data System Version 2.1: 2019 Update of Prostate Imaging Reporting and Data System Version 2. Eur Urol. 2019;76(3):340–351. 10.1016/j.eururo.2019.02.033 30898406

[3] Weinreb JC, Barentsz JO, Choyke PL, Cornud F, Haider MA, Macura KJ, et al. PI-RADS Prostate Imaging—Reporting and Data System: 2015, Version 2. Eur Urol. 2016 1;69(1):16–40. 10.1016/j.eururo.2015.08.052 26427566 PMC6467207

[4] Scialpi M, Rondoni V, Aisa MC, Martorana E, D’Andrea A, Malaspina CM, et al. Is contrast enhancement needed for diagnostic prostate MRI? Transl Androl Urol. 2017 6;6(3):499–509. 10.21037/tau.2017.05.31 28725592 PMC5503975

[5] Barentsz JO, Richenberg J, Clements R, Choyke P, Verma S, Villeirs G, et al. European Society of Urogenital Radiology. ESUR prostate MR guidelines 2012. Eur Radiol. 2012 4;22(4):746–57. 10.1007/s00330-011-2377-y 22322308 PMC3297750

[6] Tofts PS. T1-weighted DCE imaging concepts: modelling, acquisition and analysis. MAGNETOM Flash 2010;3:30–39.

[7] Mottet N, van den Bergh RCN, Briers E, Cornford P, De Santis M, Fanti S, et al. EAU Guidelines: Prostate Cancer 2019. https://uroweb.org/guideline/prostate-Cancer/ (accessed 01 Apr. 2020).

[8] Haas GP, Delongchamps N, Brawley OW, Wang CY, de la Roza G. The worldwide epidemiology of prostate cancer: perspectives from autopsy studies. Can J Urol. 2008 2;15(1):3866–71. 18304396 PMC2706483

[9] Johnson LM, Turkbey B, Figg WD, Choyke PL. Multiparametric MRI in prostate cancer management. Nat Rev Clin Oncol. 2014;11(6):346–353. 10.1038/nrclinonc.2014.69 24840072 PMC6330110

[10] Arsov C, Rabenalt R, Blondin D, Quentin M, Hiester A, Godehardt E, et al. Prospective randomized trial comparing magnetic resonance imaging (MRI)-guided in-bore biopsy to MRI-ultrasound fusion and transrectal ultrasound-guided prostate biopsy in patients with prior negative biopsies. Eur Urol. 2015 10;68(4):713–20. 10.1016/j.eururo.2015.06.008 26116294

[11] Quentin M, Blondin D, Arsov C, Schimmöller L, Hiester A, Godehardt E, et al. Prospective evaluation of magnetic resonance imaging guided in-bore prostate biopsy versus systematic transrectal ultrasound guided prostate biopsy in biopsy naïve men with elevated prostate specific antigen. J Urol. 2014 11;192(5):1374–9. 10.1016/j.juro.2014.05.090 24866597

[12] Schimmöller L, Blondin D, Arsov C, Rabenalt R, Albers P, Antoch G, et al. MRI-Guided In-Bore Biopsy: Differences Between Prostate Cancer Detection and Localization in Primary and Secondary Biopsy Settings. AJR Am J Roentgenol. 2016 1;206(1):92–9. 10.2214/AJR.15.14579 26700339

[13] Weinmann HJ, Laniado M, Mützel W. Pharmacokinetics of GdDTPA/dimeglumine after intravenous injection into healthy volunteers. Physiol Chem Phys Med NMR. 1984;16(2):167–72. . 6505043

[14] Epstein JI, Egevad L, Amin MB, Delahunt B, Srigley JR, Humphrey PA; Grading Committee. The 2014 International Society of Urological Pathology (ISUP) Consensus Conference on Gleason Grading of Prostatic Carcinoma: Definition of Grading Patterns and Proposal for a New Grading System. Am J Surg Pathol. 2016 2;40(2):244–52. 10.1097/PAS.0000000000000530 26492179

[15] Tan CH, Hobbs BP, Wei W, Kundra V. Dynamic contrast-enhanced MRI for the detection of prostate cancer: meta-analysis. AJR Am J Roentgenol. 2015;204(4):W439–W448. 10.2214/AJR.14.13373 25794093 PMC5152763

[16] Chesnais AL, Niaf E, Bratan F, Mège-Lechevallier F, Roche S, Rabilloud M, et al. Differentiation of transitional zone prostate cancer from benign hyperplasia nodules: evaluation of discriminant criteria at multiparametric MRI. Clin Radiol. 2013 6;68(6):e323–30. 10.1016/j.crad.2013.01.018 23528164

[17] Hansford BG, Peng Y, Jiang Y, Vannier MW, Antic T, Thomas S, et al. Dynamic Contrast-enhanced MR Imaging Curve-type Analysis: Is It Helpful in the Differentiation of Prostate Cancer from Healthy Peripheral Zone? Radiology. 2015 5;275(2):448–57. 10.1148/radiol.14140847 25559231

[18] Sanz-Requena R, Prats-Montalbán JM, Martí-Bonmatí L, Alberich-Bayarri Á, García-Martí G, Pérez R, et al. Automatic individual arterial input functions calculated from PCA outperform manual and population-averaged approaches for the pharmacokinetic modeling of DCE-MR images. J Magn Reson Imaging. 2015 8;42(2):477–87. 10.1002/jmri.24805 25410482

[19] Ocak I, Bernardo M, Metzger G, Barrett T, Pinto P, Albert PS, et al. Dynamic contrast-enhanced MRI of prostate cancer at 3 T: a study of pharmacokinetic parameters. AJR Am J Roentgenol. 2007 10;189(4):849. 10.2214/AJR.06.1329 17885055

[20] Mehrabian H, Da Rosa M, Haider MA, Martel AL. Pharmacokinetic analysis of prostate cancer using independent component analysis. Magn Reson Imaging. 2015 12;33(10):1236–1245. 10.1016/j.mri.2015.08.009 26297961

[21] Bonekamp D, Macura KJ. Dynamic contrast-enhanced magnetic resonance imaging in the evaluation of the prostate. Top Magn Reson Imaging. 2008;19(6):273–284. 10.1097/RMR.0b013e3181aacdc2 19512849

[22] Ahmed HU, El-Shater Bosaily A, Brown LC, Gabe R, Kaplan R, Parmar MK, et al. Diagnostic accuracy of multi-parametric MRI and TRUS biopsy in prostate cancer (PROMIS): a paired validating confirmatory study. Lancet. 2017;389(10071):815–822. 10.1016/S0140-6736(16)32401-1 28110982

[23] Niu XK, Chen XH, Chen ZF, Chen L, Li J, Peng T. Diagnostic Performance of Biparametric MRI for Detection of Prostate Cancer: A Systematic Review and Meta-Analysis. AJR Am J Roentgenol. 2018 8;211(2):369–378. 10.2214/AJR.17.18946 29894216

[24] Scialpi M, Aisa MC, D’Andrea A, Martorana E. Simplified Prostate Imaging Reporting and Data System for Biparametric Prostate MRI: A Proposal. AJR Am J Roentgenol. 2018;211(2):379–382. 10.2214/AJR.17.19014 29894218

[25] Sun Y, Reynolds HM, Wraith D, Williams S, Finnegan ME, Mitchell C, et al. Automatic stratification of prostate tumour aggressiveness using multiparametric MRI: a horizontal comparison of texture features. Acta Oncol. 2019 8;58(8):1118–1126. 10.1080/0284186X.2019.1598576 30994052

[26] Afshari Mirak S, Mohammadian Bajgiran A, Sung K, Asvadi NH, Markovic D, Felker ER, et al. Dynamic contrast-enhanced (DCE) MR imaging: the role of qualitative and quantitative parameters for evaluating prostate tumors stratified by Gleason score and PI-RADS v2. Abdom Radiol (NY). 2020 7;45(7):2225–2234. 10.1007/s00261-019-02234-6 31549211 PMC10262973

